# Accurate Classification of Differential Expression Patterns in a Bayesian Framework With Robust Normalization for Multi-Group RNA-Seq Count Data

**DOI:** 10.1177/1177932219860817

**Published:** 2019-07-08

**Authors:** Takayuki Osabe, Kentaro Shimizu, Koji Kadota

**Affiliations:** 1 Graduate School of Agricultural and Life Sciences, The University of Tokyo, Tokyo, Japan; 2 Collaborative Research Institute for Innovative Microbiology, The University of Tokyo, Japan

**Keywords:** RNA-seq, expression patterns, differential expression analysis, normalization, empirical Bayes

## Abstract

Empirical Bayes is a choice framework for differential expression (DE) analysis for multi-group RNA-seq count data. Its characteristic ability to compute posterior probabilities for predefined expression patterns allows users to assign the pattern with the highest value to the gene under consideration. However, current Bayesian methods such as baySeq and EBSeq can be improved, especially with respect to normalization. Two *R* packages (baySeq and EBSeq) with their default normalization settings and with other normalization methods (MRN and TCC) were compared using three-group simulation data and real count data. Our findings were as follows: (1) the Bayesian methods coupled with TCC normalization performed comparably or better than those with the default normalization settings under various simulation scenarios, (2) default DE pipelines provided in TCC that implements a generalized linear model framework was still superior to the Bayesian methods with TCC normalization when overall degree of DE was evaluated, and (3) baySeq with TCC was robust against different choices of possible expression patterns. In practice, we recommend using the default DE pipeline provided in TCC for obtaining overall gene ranking and then using the baySeq with TCC normalization for assigning the most plausible expression patterns to individual genes.

## Introduction

RNA-seq is a common tool to obtain expression data.^1,2^ It is popularly applied to identify differentially expressed genes (DEGs) or transcripts under different groups or conditions.^3,4^ Accurate identification of these DEGs is crucial for multiple purposes; for example, they may serve as potential biomarkers for clinical diagnosis.^5,6^ So far, many methods have been developed for analysis of RNA-seq data^7–20^ and several evaluation studies have also been performed.^21–25^ Of these, two *R*/Bioconductor^26,27^ packages—edgeR^8^ and DESeq2^9^—have been widely used for differential expression (DE) analysis of RNA-seq data.

The two packages commonly employ a generalized linear model (GLM) framework. When comparing a multi-group data (eg G1 vs G2 vs G3), the main output is an analysis of variance (ANOVA)-like *p*-value, where a low *p*-value for a gene indicates a high degree of DE in at least one of the groups compared. In other words, the output itself does not tell us which group is differentially expressed compared with the others. To confirm where the difference occurred between the three groups, for example, GLM users have to perform three two-group comparisons (ie G1 vs G2, G1 vs G3, and G2 vs G3) as a *post hoc* test and then decide the DE patterns across groups. In case of the three-group comparison, a total of five possible patterns (one non-DE pattern and four DE patterns) can be considered: G1 = G2 = G3, G1 ≠ G2 = G3, G2 ≠ G1 = G3, G3 ≠ G1 = G2, and G1 ≠ G2 ≠ G3.

However, constructing a complete expression pattern based on the results of three two-group comparisons can be difficult. For example, two possible patterns (G1 ≠ G2 = G3 or G1 = G2 = G3) can be constructed if the results of the three two-group comparisons were G1 ≠ G2, G1 = G3, and G2 = G3. Furthermore, results of the three two-group comparison themselves (ie G1 ≠ G2, G1 = G3, and G2 = G3) can vary depending on both the multiple comparison procedure and the significance level. As the number of groups to be compared increases, construction of the complete expression pattern across all groups based on the GLM framework becomes more difficult.

Different from the GLM framework, an empirical Bayesian framework implemented in baySeq^10^ and EBSeq^12^ returns one posterior probability (PP) for each of the predefined expression pattern for each gene; thus, when considering a number of patterns for a particular gene, it can assign the pattern with the highest PP to the gene under consideration. In other words, the Bayesian framework does not require subsequent analysis such as the *post hoc* test to construct the pattern. Therefore, baySeq and EBSeq provide a dedicated means for pattern classification.

We here focus on the improvement of the Bayesian framework. We demonstrate that a robust normalization strategy provided in TCC^13^ can be adapted in the analysis pipeline. Although the Bayesian framework is generally inferior to the GLM framework when the overall degree of DE is evaluated,^21,22^ accurate assignment of expression patterns to individual genes obtained from the Bayesian framework with TCC can be independently valuable.

## Materials and Methods

All analyses were performed using *R* (version 3.5.1)^26^ and Bioconductor.^27^ The versions of major *R* packages were TCC, version 1.20.1; edger, version 3.22.4; DESeq2, version 1.20.0; baySeq, version 2.14.0; EBSeq, version 1.20.0, and receiver operating characteristic (ROC) curve, version 1.6.3. The *R* codes for obtaining the current results are given in our website (http://www.iu.a.u-tokyo.ac.jp/~kadota/Osabe_2019/).

### DE analysis pipelines

In general, DE analysis consists of two steps (data normalization *X* and DEG identification *Z*), and each method has the original *X*-*Z* pipeline.^13^ The TCC package^13^ implements a multi-step normalization procedure, originally proposed by Kadota et al.^28^ The key concept is to alleviate the negative effect of potential DEGs before calculating the normalization factors. The analysis pipeline can be described as *X*-(*Y*-*X*)_*n*_ -*Z* in which *X*-(*Y*-*X*)_*n*_ corresponds to the multi-step normalization and *Y* corresponds to a DEG-identification method that may be the same as *Z*. By repeating the DEG elimination strategy in *X*-(*Y*-*X*)_*n*_, we can use accurate normalization factors at the last step *Z* in the pipeline *X*-(*Y*-*X*)_*n*_ -*Z*.

According to the previous notation,^22^ we refer to this pipeline as *XYX*-*Z* with the recommended number of *n* (=3) for short. In this case, the first three letters *XYX* correspond to the multi-step normalization. We do not use the full pipeline *XYX*-*Z* in TCC here. This is because, similar to the main output of GLM, the main output of TCC is also *p*-value that does not tell us which group is differentially expressed compared with the others.

The DE analysis typically starts with a so-called “count matrix,” where each row indicates the gene, each column indicates the sample, and each cell indicates the number of reads mapped to the gene in the sample. The *R* packages used here commonly manipulate this type of data as input. For the possible multi-step normalization procedures *XYX* in TCC, we evaluated two representatives: one is *EEE*, which consists of default methods implemented in edgeR (abbreviated as *E*) and the other is *SSS*, which consists of default methods implemented in DESeq2 (abbreviated as *S*). Specifically, *EEE* is the default procedure in TCC for multi-group data with replicates. Although two other possible procedures *XYX* in TCC (ie *SES* and *ESE*) could be evaluated, it is known that the normalization factors obtained by *SES* and *ESE* are almost the same as those obtained by *EEE* and *SSS*.^22^

For the default normalization methods *X*, two packages (edgeR and baySeq) use the TMM method^29^ and the other two packages (DESeq2 and EBSeq) use the median ratio method.^7^ The TMM method and the median ratio method correspond to *E* and *S*, respectively. Accordingly, the default DE pipeline *X*-*Z* in baySeq and EBSeq can also be abbreviated as *E*-*baySeq* and *S*-*EBSeq*, respectively. In addition to the four normalization methods (*E, S, EEE*, and *SSS*), we also evaluated another normalization method called MRN (abbreviated as *M*).^14^ Therefore, this study basically compares a total of eight DE pipelines: *EEE*-*baySeq, SSS*-*baySeq, E*-*baySeq, M*-*baySeq, EEE*-*EBSeq, SSS*-*EBSeq, S*-*EBSeq*, and *M*-*EBSeq*.

### Simulation data

In this study, to perform the multi-group comparison as simply as possible, we focused on the three-group data (G1 vs G2 vs G3) with equal numbers of biological replicates (ie 3 or 9 replicates per group; *N*_rep_ = 3 or 9). When the RNA-seq count data are based on biological replicates, the negative binomial (NB) distribution is generally applicable.^7-10^ In the NB distribution, the variance (*V*) can be modeled as $V=\mu +\varphi \mu^{2}$. The empirical distribution of read counts for producing the mean (*µ*) and dispersion (φ) parameters of the model was obtained from Arabidopsis data (three biological replicates for both the treated and nontreated samples) by Di et al.^30^

The simulation framework and evaluation metric are the same as our previous study,^22^ enabling the comparison of the current results with the previous ones. The simulation conditions were as follows: the total number of genes was 10 000 (*N*_gene_ = 10 000), 5 or 25% of the genes were DEGs (*P*_DEG_ = 0.05 or 0.25), the DE levels were four-fold in individual groups, and the proportions of up-regulated DEGs in individual groups (*P*_G1_, *P*_G2_, *P*_G3_) were (1/3, 1/3, 1/3), (0.5, 0.3, 0.2), (0.5, 0.4, 0.1), (0.6, 0.2, 0.2), (0.6, 0.3, 0.1), (0.7, 0.2, 0.1), and (0.8, 0.1, 0.1). The shape of the distribution for introduced DEGs is the same as that of non-DEGs. The *simulateReadCounts* function provided in TCC was used to generate three-group simulation data. The output of the *simulateReadCounts* function is stored in the TCC class object with information about the simulation conditions and is therefore ready-to-analyze.

### Real data

A total of three count data sets were analyzed. The first data set was originally sequenced from the three species (ie the three-group data): humans (G1), chimpanzees (G2), and rhesus macaques (G3).^31^ Briefly, Blekhman et al studied expression levels of liver samples from three males and three females from each species, giving a total of six different individuals (ie six biological replicates) for each species. Since they performed duplicate experiments for each individual (ie two technical replicates), the publicly available raw count matrix consists of 20 689 genes × 36 samples (=3 species × 6 biological replicates × 2 technical replicates). To correctly estimate the biological variation and make the assumed structure of input data, we summed and collapsed the count data of technical replicates, giving a reduced number of columns in the count matrix (ie 18 samples).

The second and third data sets were derived from a study of human brain (SRP056477).^32^ The count data consisting of 58 037 genes × 52 samples were obtained from the recount2 database.^33^ The samples were divided into two source types: 25 cerebellum (CER) and 27 frontal cortex (FCX) samples, and further subdivided into three case types: healthy, sporadic amyotrophic lateral sclerosis (sALS), and ALS caused by a repeat expansion in C9orf72 (c9ALS). Accordingly, we performed two three-group comparisons (G1 vs G2 vs G3 as healthy vs sALS vs c9ALS) for these data. For simplicity, we excluded one sample (SRR1927053) from the CER data set and three samples (SRR1927071, SRR1927052, and SRR1927054) from the FCX data set such that each group had eight replicates.

### Normalization

A total of five normalization methods (*E, S, EEE, SSS*, and *M*) were evaluated. The *E* was calculated using the *getLibsizes* function with the option (*estimationType* *=* “*edger*”) in baySeq. The *S* was calculated using the *MedianNorm* function with default option in EBSeq. The *EEE* was calculated using the *calcNormFactors* function with options (*norm.method* *=* “*tmm*” and *test.method* *=* “*edger*”) in TCC. The *SSS* was calculated using the *calcNormFactors* function with options (*norm.method* *=* “*deseq2*” and *test.method* *=* “*deseq2*”) in TCC. The *M* was calculated using the *mrnFactors* function provided by Maza.^34^

### Expression patterns

In this study, a total of five possible expression patterns were considered when performing the Bayesian methods: non-DEG pattern (say “*nonDEG*”), DE pattern up-regulated or down-regulated in G1 (*DEG_G1*), DE pattern in G2 (*DEG_G2*), DE pattern in G3 (*DEG_G3*), and DE pattern between all groups (*DEG_all*). In simulation analysis, only the first four patterns (ie *nonDEG, DEG_G1, DEG_G2*, and *DEG_G3*) were considered.

### DE analysis with baySeq

The baySeq was performed using the *getPriors.NB* function with options (*samplesize* *=* *2000* and *estimation* *=* “*QL*”) and then the *getLikelihoods* function with options, *pET* *=* “Bayesian information criterion (BIC)” and *nullData* *=* *FALSE*. The PPs assigned for *nonDEG* were used to rank genes. Genes with *q*-value < 0.05 (ie 5% nominal false discovery rate (FDR) threshold) were regarded as DEG. Expression patterns for genes with *q*-value ⩾ 0.05 were regarded as *nonDEG*.

### DE analysis with EBSeq

The EBSeq was performed using the *EBMultiTest* function with options (*maxround* *=* *5, Qtrm* *=* *1.0*, and *QtrmCut* *=* *–1*) and then the *GetMultiPP* function. The PPs assigned for *nonDEG* were used to rank genes. Genes with *q*-value < 0.05 (ie 5% nominal FDR threshold) were regarded as DEG. Expression patterns for genes with *q*-value ⩾ 0.05 were regarded as *nonDEG*.

### Evaluation metrics

The evaluation was performed using the rank information of PPs assigned for *nonDEG*. The area under the ROC curve (AUC), which evaluates both sensitivity and specificity of the DE pipelines simultaneously, was used as a main measure of comparison. A good pipeline has a high AUC value (ie high sensitivity and specificity). Two input vectors are required to calculate the AUC value. We used two numeric vectors as input: one was the rank information obtained from the DE pipeline and the other was the binary information indicating which gene is non-DEG (0) or DEG (1). The two functions (*rocdemo.sca* and *AUC*) provided in the ROC package was used to calculate the AUC value.

In case of the binary (0 for non-DEG and 1 for DEG) classification problem, genes predicted as DEG by the pipeline were labeled as either true positive (TP) or false positive (FP). Genes labeled as TP correspond to those correctly predicted as DEG (ie the truth is DEG) and genes labeled as FP correspond to those falsely predicted as DEG (ie the truth is non-DEG). Similarly, genes predicted as non-DEG by the pipeline can be labeled as either true negative (TN) or false negative (FN). Genes labeled as TN correspond to those correctly predicted as non-DEG (ie the truth is non-DEG) and genes labeled as FN correspond to those falsely predicted as non-DEG (ie the truth is DEG). The accuracy was calculated as (TP + TN)/(TP + TN + FP + FN), where the denominator corresponds to the total number of genes (=10 000) and the numerator corresponds to the total number of correctly predicted genes. The actual FDR, sensitivity, and specificity were calculated as FP/(FP + TP), TP/(TP + FN), and TN/(TN + FP), respectively.

In this study, the Bayesian methods (baySeq and EBSeq) were performed by considering four or five possible expression patterns. When considering four expression patterns (ie *nonDEG, DEG_G1, DEG_G2*, and *DEG_G3*), for example, the numerator of the equation for calculating the accuracy was defined as the total number of correctly predicted genes for individual patterns. Only genes correctly predicted in each of the patterns were counted.

## Results and Discussion

### Simulation results when considering four expression patterns (N_rep_ = 3)

We assessed the performance of a total of eight DE pipelines when four possible expression patterns (*nonDEG, DEG_G1, DEG_G2*, and *DEG_G3*) were considered. Table 1 lists the average AUC values of 100 trials for simulation data with three replicates per group (ie *N*_rep_ = 3). Overall, the four baySeq-related pipelines (*EEE-baySeq, SSS-baySeq, E-baySeq*, and *M-baySeq*) outperformed the four EBSeq-related pipelines (*EEE-EBSeq, SSS-EBSeq, E-EBSeq*, and *M-EBSeq*). This is probably because the baySeq model is closer to the simulation data than the EBSeq model. In this sense, it may be better to compare between only four DE pipelines using the same package.

**Table 1.** Average AUC values for simulation data (*N*_rep_ = 3).

*P*_G1_	1/3	0.5	0.5	0.6	0.6	0.7	0.8
*P*_G2_	1/3	0.3	0.4	0.2	0.3	0.2	0.1
*P*_G3_	1/3	0.2	0.1	0.2	0.1	0.1	0.1
(a) *P*_DEG_ = 0.05							
* EEE-baySeq*	90.38	90.43	**90.45**	**90.48**	**90.46**	**90.47**	90.56
* SSS-baySeq*	90.37	90.39	90.41	90.43	90.43	90.46	**90.58**
* E-baySeq*	90.38	90.39	90.38	90.38	90.37	90.37	90.45
* M-baySeq*	**90.41**	**90.45**	90.40	90.40	90.42	90.44	90.52
* EEE-EBSeq*	85.77	85.87	85.83	85.87	85.79	85.82	85.93
* SSS-EBSeq*	85.78	85.88	85.83	85.86	85.80	85.81	85.94
* S-EBSeq*	85.78	85.86	85.79	85.82	85.73	85.72	85.78
* M-EBSeq*	85.73	85.83	85.77	85.82	85.75	85.74	85.85
(b) *P*_DEG_ = 0.25							
* EEE-baySeq*	90.38	90.38	**90.48**	90.48	90.49	90.63	90.76
* SSS-baySeq*	90.35	**90.39**	**90.48**	**90.49**	**90.50**	**90.66**	**90.77**
* E-baySeq*	90.38	90.23	90.23	90.03	90.00	89.71	89.01
* M-baySeq*	**90.40**	90.34	90.38	90.31	90.27	90.21	89.95
* EEE-EBSeq*	85.82	85.88	85.92	85.94	85.97	85.95	86.06
* SSS-EBSeq*	85.82	85.87	85.94	85.95	85.99	85.99	86.13
* S-EBSeq*	85.83	85.63	85.51	85.35	85.31	84.75	84.02
* M-EBSeq*	85.78	85.76	85.76	85.70	85.71	85.48	85.20

Abbreviation: AUC, area under the ROC curve. Average AUC values (%) of 100 trials for each simulation condition are shown: (a) *P*_DEG_ = 0.05 and (b) *P*_DEG_ = 0.25. A total of seven conditions are shown. The highest AUC values for each condition are in bold.

**Figure 1.**
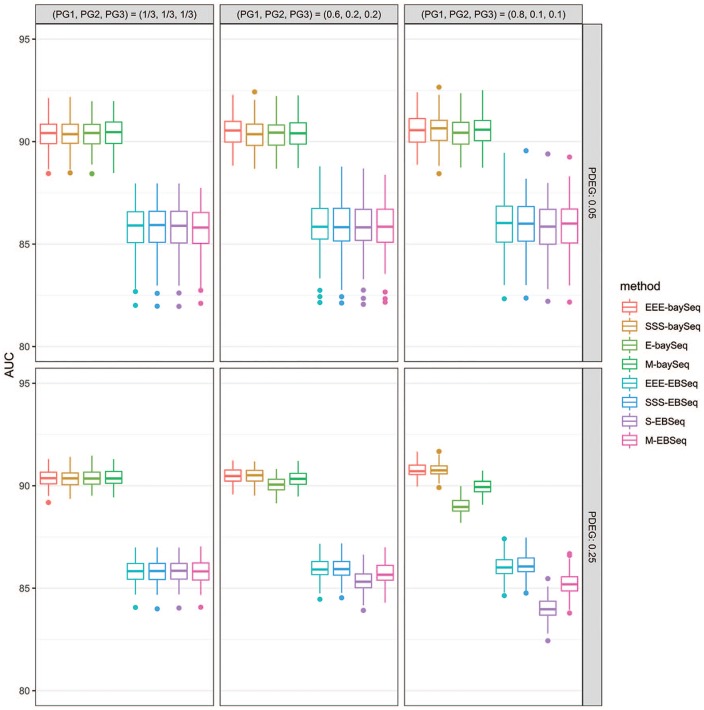
Boxplots of AUC values for simulation data (*N_rep_* = 3). Abbreviation: AUC, area under the ROC curve. Representative boxplots for AUC values under three conditions, (1/3, 1/3, 1/3), (0.6, 0.2, 0.2), and (0.8, 0.1, 0.1), are shown. The average AUC values in the plots are shown in Table 1.

When we compared four baySeq-related pipelines, the AUC values for the baySeq with TCC (ie *EEE-baySeq* and *SSS-baySeq*) were the highest and similar across the seven conditions. Relative performances for the default baySeq pipeline (ie *E-baySeq*) compared with those for *XYX-baySeq* generally worsened as the degrees of biases increased (ie from left to right in Table 1). The trend was more pronounced when a higher amount of DEGs was introduced (ie *P*_DEG_ = 25%; Table 1). Figure 1 shows representative boxplots of the AUC values under three conditions. The differences between *XYX-baySeq* and *E-baySeq* can be clearly seen at both the most biased condition (0.8, 0.1, 0.1) and *P*_DEG_ = 25%. We confirmed the superiority of *XYX-baySeq* by using another evaluation metric (ie accuracy; see Sheet 2 in Additional file 1 of Supplemental material).

Surprisingly, *M-baySeq* consistently outperformed *E-baySeq* under the simulation conditions investigated, despite the theoretical similarity between the two normalization methods (ie *M* and *E*).^34^ A multi-step normalization procedure based on MRN (ie iterative MRN or *MMM*) can be constructed in principle. The performance for baySeq with *MMM* (ie *MMM-baySeq*) could be similar or higher to those for baySeq with TCC (ie *EEE-baySeq* and *SSS-baySeq*).

While the AUC values shown in Table 1 were comparable to those in our previous study (ie Table 1 by Tang et al^22^), the values for *E-baySeq* and *S-EBSeq* were slightly different from those observed in previous studies. This can be explained by the difference in the possible patterns considered. While Table 1 considered four patterns (*nonDEG, DEG_G1, DEG_G2*, and *DEG_G3*), the previous study considered two (*nonDEG* and *DEG_all*) and five patterns (*nonDEG, DEG_G1, DEG_G2, DEG_G3*, and *DEG_all*) when performing baySeq and EBSeq, respectively.

Despite the different numbers of possible patterns to be considered, we observed similar AUC values between the current and previous studies. This is probably because these values are calculated based on the PPs assigned for the *nonDEG* pattern; the AUC value is used as a measure of the discriminability between non-DEG and the others. In other words, a high AUC value does not necessarily indicate a high classification performance within DEG. Nevertheless, we observed the superiority of *XYX-baySeq* to *X-baySeq* in terms of the classification performance within the three kinds of DEGs (see Sheet 4 in Additional file 1 of Supplemental material).

In practice, it is important to control FDR when identifying DEGs. Remember we regarded genes satisfying *q*-value < 0.05 (ie 5% nominal FDR) as DEGs when constructing the confusion matrix (Sheet 4 in Additional file 1 of Supplemental material). We investigated the actual FDR under the nominal value (Sheet 5 in Additional file 1 of Supplemental material). The baySeq-related pipelines had consistently better values in terms of the actual FDR than the EBSeq-related pipelines.

It should be noted that the AUC values (<91%) of the best performing pipeline (ie *XYX-baySeq*) shown in Table 1 are consistently lower than those (>91%) of the full analysis pipeline provided in TCC (ie *EEE*-*E* shown in Table 1 by Tang et al^22^). This fact indicates that the *EEE*-*E* as a non-Bayesian DE pipeline can be recommended when the overall degree of DE is evaluated. However, as described previously, the primary output of *EEE-E* does not tell us which group is differentially expressed compared with the others. While it is possible to roughly know the DE group(s) in a GLM framework by, for example, performing all pairs of two-group comparisons, constructing a complete expression pattern across all groups can be difficult. Indeed, two common packages (edgeR and DESeq2) as well as TCC, which implement GLM framework, do not provide an approach for obtaining the complete expression pattern for each gene. Therefore, our focus on improving the Bayesian framework makes sense.

### Simulation results when considering four expression patterns (N_rep_ = 9)

We previously reported that the relative performances for the original EBSeq pipeline (ie *S-EBSeq*) tend to improve as the *N*_rep_ increases.^22^ We performed a similar analysis, as shown in Table 1, with *N*_rep_ = 9 (Additional file 2 of Supplemental material). Although the AUC values for the two *XYX-EBSeq* pipelines (ie *EEE-EBSeq* and *SSS-EBSeq*) were the highest overall and similar across the seven different conditions, the superiority was not observed when evaluating the accuracy (Sheetd2 in Additional file 2 of Supplemental material). This can be explained by the high actual FDR (>33% at *P*_DEG_ = 0.25 and >16% at *P*_DEG_ = 0.05) compared with the nominal value (=5%). Nevertheless, it can be said that the Bayesian methods with TCC are promising for accurate classification of DE patterns.

### Simulation results when considering five expression patterns

Remember that the simulation data have four expression patterns (*nonDEG, DEG_G1, DEG_G2*, and *DEG_G3*). However, another possible pattern (ie *DEG_all*) can practically be considered for the three-group comparison. To investigate the effect of *DEG_all*, we performed the Bayesian-based methods with five possible expression patterns. The results with *N*_rep_ = 3 and 9 are shown in Additional files 3 and 4 of Supplemental material, respectively.

Overall, the number of genes assigned to the *DEG_all* pattern was small (ie low FPs). In particular, the *XYX-baySeq* pipelines showed very few *DEG_all* genes (maximum 0.02%; see Sheet 4 in Additional files 3 and 4 of Supplemental material). This is mainly because baySeq tends to detect fewer DEGs than EBSeq. Indeed, *EEE-baySeq* and *EEE-EBSeq* called 1993.32 and 2698.48 DEGs satisfying 5% nominal FDR, respectively, under the simulation condition: *N*_rep_ = 9, *N*_gene_ = 10 000, *P*_DEG_ = 0.25, *P*_G1_ = 0.8, *P*_G2_ = 0.1, and *P*_G3_ = 0.1. The truth for this condition is 10 000 × 0.25 = 2500 DEGs. This characteristic of baySeq results in low FPs (ie low Type I error) as well as low TPs, leading to high precision with low sensitivity. Similarly, EBSeq results in high FPs as well as high TPs (ie low Type II error), leading to low precision with high sensitivity. There is a trade-off between Type I and Type II errors. Nevertheless, the conclusions derived from the case of four patterns remained unchanged for the case of five patterns.

### Results for real data

We analyzed a real data set consisting of 20 689 genes × 18 liver samples for three-group comparison: six humans (G1), six chimpanzees (G2), and six rhesus macaques (G3).^31^ Table 2 shows the number of genes assigned to individual patterns when considering (1) five patterns, (2) four patterns, and (3) the common ones. Similar to the simulation results, the numbers of genes assigned to *DEG_all* were relatively small (Table 2). In particular, baySeq had fewer identified DEGs overall, and thus, the influence of the presence or absence of *DEG_all* pattern was less than that of EBSeq.

**Table 2.** Numbers of genes assigned to individual patterns.

DE pipeline	*nonDEG*	*DEG_G1*	*DEG_G2*	*DEG_G3*	*DEG_all*	Total
(a) Five possible expression patterns						
* EEE-baySeq*	15 106	914	877	3473	319	20 689
* SSS-baySeq*	14 999	938	893	3539	320	20 689
* E-baySeq*	15 032	935	886	3507	329	20 689
* M-baySeq*	15 400	884	805	3298	302	20 689
* EEE-EBSeq*	12 798	1284	1455	4471	681	20 689
* SSS-EBSeq*	12 782	1302	1430	4476	699	20 689
* S-EBSeq*	12 821	1253	1442	4474	699	20 689
S-EBSeq*	12 826	1253	1439	4482	689	20 689
* M-EBSeq*	13 042	1198	1368	4435	646	20 689
(b) Four possible expression patterns						
* EEE-baySeq*	14 986	1026	978	3699	−	20 689
* SSS-baySeq*	14 983	1031	958	3717	−	20 689
* E-baySeq*	14 978	1037	976	3698	−	20 689
* M-baySeq*	15 330	985	890	3484	−	20 689
* EEE-EBSeq*	9866	1517	1665	7641	−	20 689
* SSS-EBSeq*	9816	1560	1648	7665	−	20 689
* S-EBSeq*	12 662	1504	1671	4852	−	20 689
S-EBSeq*	9872	1502	1666	7649	−	20 689
* M-EBSeq*	12 842	1426	1606	4815	−	20 689
(c) Common						
* EEE-baySeq*	14 890	887	846	3405	−	20 028
* SSS-baySeq*	14 849	898	851	3464	−	20 062
* E-baySeq*	14 844	896	842	3424	−	20 006
* M-baySeq*	15 205	846	769	3220	−	20 040
* EEE-EBSeq*	9865	1284	1455	4445	−	17 049
* SSS-EBSeq*	9815	1302	1430	4448	−	16 995
* S-EBSeq*	12 661	1253	1441	4446	−	19 801
S-EBSeq*	9871	1253	1438	4452	−	17 014
* M-EBSeq*	12 841	1198	1368	4404	−	19 811

Abbreviations: DE, differential expression; FDR, false discovery rate. Genes satisfying 5% nominal FDR (*q*-value < 0.05) were regarded as DEG. In the original EBSeq pipeline, *S*-*EBSeq* was performed using Bayesian method with size factors, and *S*-EBSeq* was performed using Bayesian method with *normalized* size factors such that the mean was 1.

Surprisingly, we observed considerably different results among the EBSeq-related pipelines with four patterns (Table 2). While three pipelines (*EEE-EBSeq, SSS-EBSeq*, and S-EBSeq*) identified much less *nonDEG* genes (<10 000), *S-EBSeq* and *M-EBSeq* identified 12 662 and 12 842 *nonDEG* genes, respectively and were similar to the results obtained with the four EBSeq-related pipelines with five patterns. The three EBSeq-related pipelines (*EEE-EBSeq, SSS-EBSeq*, and S-EBSeq*) commonly employ *normalized* size factors. In particular, the difference between S-EBSeq* and *S-EBSeq* is only the presence or absence of size factor normalization; the mean values of size factors before and after normalization were 1.027 and 1.000, respectively. It is difficult to understand that this slight difference of 0.027 affected the four but not the five patterns. We did not observe this phenomenon for the simulation data and another real data (Additional file 5 of Supplemental material).^32^ To the best of our knowledge, this is the first study to report that the presence or absence of size factor normalization can have a large effect on DE result when using EBSeq.

## Conclusions

We evaluated a total of eight Bayesian-based DE pipelines using three-group RNA-seq count data. The Bayesian methods coupled with TCC normalization performed comparably or better than the Bayesian methods with the default normalization settings. In particular, the AUC values for *XYX-baySeq* (ie *EEE-baySeq* and *SSS-baySeq*) were higher overall than the other Bayesian-based pipelines. Since *EEE* is the default normalization method of TCC, using *EEE-baySeq* would be the best practice among the eight Bayesian-based pipelines interrogated. We also confirmed that *EEE-baySeq* was robust against different choices of possible expression patterns. It is useful to obtain complete expression pattern across all groups compared, by taking advantage of the Bayesian framework.

It is important to note that we do not recommend the use of *EEE-baySeq* if the purpose is to rank genes according to the overall degree of DE. This is because the AUC values for the default DE pipeline of TCC (ie *EEE-E*) were still higher than those for *EEE-baySeq*. Therefore, the use of *EEE-E* can be recommended for the purpose. In practice, the *EEE-baySeq* is useful as a subsequent analysis of *EEE-E*. By assigning the most plausible expression patterns to individual genes that are ranked according to the overall degree of DE, an accurate classification would be accomplished.

## Supplemental Material

Additional1_xyz19222377f73da – Supplemental material for Accurate Classification of Differential Expression Patterns in a Bayesian Framework With Robust Normalization for Multi-Group RNA-Seq Count Data Click here for additional data file. Supplemental material, Additional1_xyz19222377f73da for Accurate Classification of Differential Expression Patterns in a Bayesian Framework With Robust Normalization for Multi-Group RNA-Seq Count Data by Takayuki Osabe, Kentaro Shimizu and Koji Kadota in Bioinformatics and Biology Insights

## Supplemental Material

Additional2_xyz1922267354467 – Supplemental material for Accurate Classification of Differential Expression Patterns in a Bayesian Framework With Robust Normalization for Multi-Group RNA-Seq Count Data Click here for additional data file. Supplemental material, Additional2_xyz1922267354467 for Accurate Classification of Differential Expression Patterns in a Bayesian Framework With Robust Normalization for Multi-Group RNA-Seq Count Data by Takayuki Osabe, Kentaro Shimizu and Koji Kadota in Bioinformatics and Biology Insights

## Supplemental Material

Additional3_xyz19222d459e214 – Supplemental material for Accurate Classification of Differential Expression Patterns in a Bayesian Framework With Robust Normalization for Multi-Group RNA-Seq Count Data Click here for additional data file. Supplemental material, Additional3_xyz19222d459e214 for Accurate Classification of Differential Expression Patterns in a Bayesian Framework With Robust Normalization for Multi-Group RNA-Seq Count Data by Takayuki Osabe, Kentaro Shimizu and Koji Kadota in Bioinformatics and Biology Insights

## Supplemental Material

Additional4_xyz1922271719133 – Supplemental material for Accurate Classification of Differential Expression Patterns in a Bayesian Framework With Robust Normalization for Multi-Group RNA-Seq Count Data Click here for additional data file. Supplemental material, Additional4_xyz1922271719133 for Accurate Classification of Differential Expression Patterns in a Bayesian Framework With Robust Normalization for Multi-Group RNA-Seq Count Data by Takayuki Osabe, Kentaro Shimizu and Koji Kadota in Bioinformatics and Biology Insights

## Supplemental Material

Additional5_xyz19222be3d8dc9 – Supplemental material for Accurate Classification of Differential Expression Patterns in a Bayesian Framework With Robust Normalization for Multi-Group RNA-Seq Count Data Click here for additional data file. Supplemental material, Additional5_xyz19222be3d8dc9 for Accurate Classification of Differential Expression Patterns in a Bayesian Framework With Robust Normalization for Multi-Group RNA-Seq Count Data by Takayuki Osabe, Kentaro Shimizu and Koji Kadota in Bioinformatics and Biology Insights
